# The Impact of Protein Structure and Sequence Similarity on the Accuracy of Machine-Learning Scoring Functions for Binding Affinity Prediction

**DOI:** 10.3390/biom8010012

**Published:** 2018-03-14

**Authors:** Hongjian Li, Jiangjun Peng, Yee Leung, Kwong-Sak Leung, Man-Hon Wong, Gang Lu, Pedro J. Ballester

**Affiliations:** 1 SDIVF R&D Centre, Hong Kong Science Park, Sha Tin, New Territories, Hong Kong, China; jackyleehongjian@gmail.com; 2 Institute of Future Cities, The Chinese University of Hong Kong, Sha Tin, New Territories, Hong Kong, China; andrew.pengjj@gmail.com (J.P.); yeeleung@cuhk.edu.hk (Y.L.); ksleung@cse.cuhk.edu.hk (K.-S.L.); 3 Department of Computer Science and Engineering, The Chinese University of Hong Kong, Sha Tin, New Territories, Hong Kong, China; mhwong@cse.cuhk.edu.hk; 4 School of Mathematics and Statistics, Xi’an Jiaotong University, Xi’an 710049, China; 5 School of Biomedical Sciences, The Chinese University of Hong Kong, Sha Tin, New Territories, Hong Kong, China; lugang@cuhk.edu.hk; 6 Cancer Research Center of Marseille, INSERM U1068, F-13009 Marseille, France; 7 Institut Paoli-Calmettes, F-13009 Marseille, France; 8 Aix-Marseille Université, F-13284 Marseille, France; 9 CNRS UMR7258, F-13009 Marseille, France

**Keywords:** machine learning, scoring function, molecular docking, binding affinity prediction

## Abstract

It has recently been claimed that the outstanding performance of machine-learning scoring functions (SFs) is exclusively due to the presence of training complexes with highly similar proteins to those in the test set. Here, we revisit this question using 24 similarity-based training sets, a widely used test set, and four SFs. Three of these SFs employ machine learning instead of the classical linear regression approach of the fourth SF (X-Score which has the best test set performance out of 16 classical SFs). We have found that random forest (RF)-based RF-Score-v3 outperforms X-Score even when 68% of the most similar proteins are removed from the training set. In addition, unlike X-Score, RF-Score-v3 is able to keep learning with an increasing training set size, becoming substantially more predictive than X-Score when the full 1105 complexes are used for training. These results show that machine-learning SFs owe a substantial part of their performance to training on complexes with dissimilar proteins to those in the test set, against what has been previously concluded using the same data. Given that a growing amount of structural and interaction data will be available from academic and industrial sources, this performance gap between machine-learning SFs and classical SFs is expected to enlarge in the future.

## 1. Introduction

A Scoring Function (SF) for structure-based protein–ligand binding affinity prediction has an essential influence on the reliability of molecular docking. Enhancing the accuracy of SFs has proven to be a challenging task for any class of method. SFs can be methodologically categorized into two broad classes: classical SFs and machine-learning SFs. Classical SFs assume a predetermined theory-inspired functional form for the relationship between the variables that characterise the complex and its predicted binding affinity and usually adopt linear regression with a small number of expert-selected structural features. On the other hand, machine-learning SFs do not impose a particular functional form for the SF. Instead, these SFs aim at implicitly capturing binding interactions that are hard to model explicitly.

The development of SFs based on modern machine-learning regression models has been a fruitful research topic in recent years [1,2,3,4,5,6,7]. These machine-learning SFs have been shown to outperform a wide range of classical SFs at the two related problems of binding affinity prediction and virtual screening [8]. Benchmarks to test SFs for binding affinity prediction are typically carried out using a crystal structure for each considered protein–ligand complex, as this experimental setup does not suffer from confounding factors such as the redocking pose error or the uncertainty of whether the molecule actually binds to the protein. Here, we investigate how the degree of similarity between proteins in training and test sets influence the performance of SFs. This question has recently been addressed by Li and Yang [9], but our expanded analysis has led to other conclusions that we report here.

Li and Yang published a study [9] intended to analyse how the use of highly similar proteins impacts the performance of SFs. More precisely, highly similar proteins were training complexes whose proteins were highly similar to those in the test complexes. The issue was investigated for classical and machine-learning SFs [8], with X-Score [10] as the classical SF and the first version of random forest (RF)-based RF-Score [1] as the machine-learning SF. They focused on the ability of these SFs to predict the binding affinities of the test protein–ligand complexes from their crystal structures (also known as their scoring power) using a common benchmark [10]. Pearson correlation (Rp) between predicted and measured affinities of the test set complexes was used to assess the scoring power of the two SFs. Each SF was trained with a series of nested training sets, ranging from small sets of highly dissimilar proteins to large sets that also include highly similar proteins (each training set is a strict subset of any larger training set).

From those experiments, Li and Yang claimed that classical SFs have the following advantages over machine-learning SFs:

- The scoring power of X-Score is stable and independent of training complexes with highly similar proteins to those in the test set, which is necessary for real-world applications.
- The outstanding scoring power of RF-Score is due to a higher number of similar proteins and not to increasing training set size.
- After removal of highly similar proteins from the training set, RF-Score does not outperform X-Score anymore.
- The reason for the improvement of the scoring power made by machine-learning SFs remains unclear.

## 2. Results

**Figure 1:**
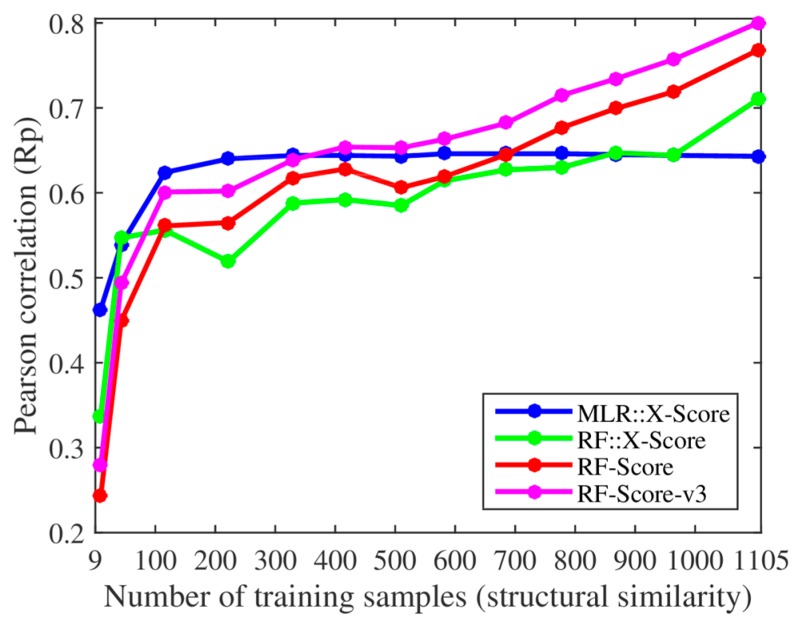
Test set performance of scoring functions (SFs) trained with nested datasets at different protein structural similarity cutoffs. Each of the four SFs (see legend at the right bottom) was trained with 13 nested datasets (i.e., a larger dataset includes all the complexes from the smaller datasets), leading to 13 implementations of each SF. This method to generate datasets was introduced by Li and Yang [9] to include training complexes with increasingly similar proteins to those in the test set (the smaller the data set is, the lower the applied protein structural similarity cutoff was). However, to look deeper into these questions, we incorporated two smaller training sets and also included in the comparison two additional machine-learning SFs (RF::X-Score and RF-Score-v3). For each SF implementation, the performance was calculated as the Pearson correlation between the predicted and the measured binding affinities for the 195 diverse protein–ligand complexes in the test set. We can see that X-Score’s performance levels off with as little as 116 training complexes, hence being unable to exploit the most similar complexes. By contrast, RF-Score-v3 keeps learning, outperforming X-Score with training sets larger than just 371 complexes. Note the large performance gap between machine-learning SFs and X-Score when the full 1105 complexes were all used for training. Abbreviations: RF, random forest; MLR, multiple linear regression.

We, however, have found several issues in the experiments employed to support these claims. First, the smallest training set already contained 116 complexes when using their protein structural similarity ranking (cutoff = 0.4), so we added two smaller training sets with only 43 and 9 complexes (cutoff = 0.35 and 0.3). Thus, we retrained both X-Score and RF-Score with 13 nested training sets, which led to the corresponding 13 test set Rp values per SF shown in Figure 1. With the addition of the two smallest training sets, it was clearer that the performance of X-Score leveled off with as little as 116 training complexes (10.5% of the employed training data), being incapable of exploiting similar proteins, whereas RF-Score was able to keep learning until surpassing X-Score with training sets larger than 700 complexes. Second, we also trained and tested the third version of RF-Score [11] (RF-Score-v3) with the same data sets (the results of RF-Score-v3 were not shown in [9]). Contrary to what Li and Yang stated in their paper [9], some conclusions did change dramatically from theirs when using this updated version of RF-Score: RF-Score-v3 obtained a better performance than X-Score with training sets larger than just 371 complexes (33.6% of the training data). It is worth nothing that these 371 complexes were the most dissimilar training samples, which means that even when trained with a moderate percent of dissimilar proteins RF-Score-v3 would already outperform X-Score. It is also worth noting the large improvement in performance achieved by RF-Score-v3 over X-Score when more training complexes were available, especially as X-Score is the SF with the best test set Rp out of 16 classical SFs [10].

**Figure 2:**
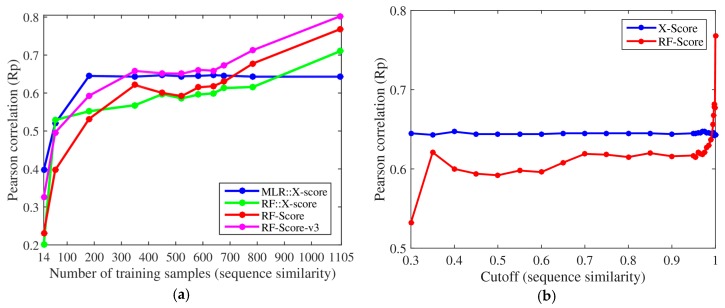
Test set performance of SFs trained with nested data sets at different protein sequence similarity cutoffs. (**a**) Instead of protein structural similarity (Figure 1), sequence similarity cutoffs were here employed to build 11 nested training sets, including two training sets smaller than those previously used in [9]. In addition, we also included in the comparison two additional machine-learning SFs (RF::X-Score and RF-Score-v3). Each of the four SFs was trained with these nested data sets, leading to 11 implementations of each SF. Each of the resulting 44 SF implementations was tested against the same 195 diverse protein–ligand complexes in the test set. Analogous conclusions were reached in this case. X-Score cannot benefit from more than 181 training samples, whereas its RF variant (denoted as RF::X-Score) and both RF-Score and RF-Score-v3 keep learning and increasing the correlation of their predicted binding affinities to the experimentally measured values and ultimately surpassed X-Score. (**b**) This plot reproduces the relevant part of the graphical abstract of Li and Yang’s paper. Note that, to label one training set complex as similar, it was enough that a single test set complex had an above-cutoff similarity to the training complex. Because the test set was generated by picking three representatives of each of the 65 sequence-based clusters, the vast majority of the remaining test complexes would tend to contain a dissimilar protein to that of the *similar* training complex.

The same conclusions were also reached using protein sequence similarity to define 11 nested training sets (left plot in Figure 2), of which the two smallest sets were new in the present study, with only 56 and 14 complexes (cutoff = 0.25 and 0.2). The performance of X-Score stagnated with merely 181 training complexes and did not improve with more similar training complexes. By contrast, RF-Score-v3 started to overtake X-Score with just 350 training complexes (31.7% of the training data). Moreover, we also evaluated a RF variant of X-Score, denoted as RF::X-Score, which employs the same features. Figure 1 and Figure 2 demonstrate that machine-learning SFs outperform classical SFs, given sufficient training samples. Even without introducing any further methodological enhancement, this performance gap will broaden as more structural and interaction data become available. For comparison, the right plot in Figure 2 reproduces the relevant part of the graphical abstract of Li and Yang’s paper [9]. Both plots in Figure 2 employ exactly the same training and test datasets (except for the two smallest training datasets with cutoffs 0.25 and 0.2, which were not considered in [9]). Without these two small datasets and the two additional machine-learning SFs and by not making explicit the number of complexes in each training set, the advantages that machine-learning SFs have over their classical counterparts are easy to miss.

Lastly, we explain why statistical significance tests have not been carried out in comparing classical SFs with machine-learning SFs. The multiple linear regression (MLR) method employed by X-Score is deterministic, i.e., training X-Score with the same data set always results in the same model. In contrast, RF is stochastic, as it involves random bootstrap sampling of the training data. Here, we built 10 instances for each machine-learning SF and reported the average performance. This is a usual procedure because the test set performance variability due to the stochastic nature of RF is much smaller than the difference in performance due to modelling choices. For instance, the 10 Rp values for RF-Score-v3 with the entire training set (1105 complexes) ranged from 0.798 to 0.803, whereas X-Score’s Rp was just 0.643 (i.e., the median Rp of RF-Score-v3, 0.800, −31.4 times its stochastic variability range of 0.005). Therefore, the *p*-values would be all extremely low and add little to the reported difference in median values.

## 3. Discussion

Regarding Claim A in Section 1 (X-Score has the advantage of being independent of highly similar proteins), Figure 1 shows that X-Score is actually unable to exploit about 89.5% of the training complexes, including those with the most similar proteins. Figure 2 shows that the same situation is encountered when using training sets generated with sequence similarity instead of structural similarity. Far from being an advantage, this means that RF-Score is substantially more predictive than X-Score, and that this performance gap will widen as more data are used for training. This is also true for RF::X-Score in Figure 1, which is a machine-learning SF generated by employing the same data and terms (features) as X-Score, but adopting RF [12] instead of the linear regression characterising classical SFs. In fact, this has also been shown to be the case for other classical SFs, which also became more predictive after they were modified in this way (e.g., AutoDock Vina [11] or Cyscore [13]). It is also worth mentioning that RF is not the only machine-learning algorithm that leads to strongly outperforming X-Score and other classical SFs, e.g., this is also the case of support vector regression (SVR) using the same features and data set as X-Score [14].

It is easy to see that Claim B (RF-Score’s performance is exclusively due to a higher number of highly similar proteins in the training set and not to larger training set sizes) does not hold. New protein–ligand complexes for training may contain proteins with any degree of similarity to those in the test set. A certain proportion of these complexes will therefore contain highly similar proteins. Consequently, increasing the training set size will also increase the number of highly similar proteins. In fact, the scoring power of machine-learning SFs has already been demonstrated to increase with the training set size elsewhere [2,11,13,15]. Furthermore, it is important to note that any decrease in performance following the removal of a training set complex similar to a test set complex might also be due to larger errors on the other 194 test complexes. Moreover, RF-Score-v3 outperformed X-Score even when the 734 complexes with the most structurally similar proteins were not included in the training set (755 of the 1105 complexes when carrying out the same experiment with sequence similarity). These results show that the large difference in test set performance was not only due to the highly similar proteins in the training set.

We are puzzled about Claim C (X-Score is better than RF-Score if we remove all the highly similar proteins from the training set): why would anyone want to remove the most relevant data for prediction? Since data from highly similar proteins are the most suitable for training SFs on this problem, it does not make any sense to remove them, and therefore RF-Score outperforms X-Score in predicting the binding affinities of a diverse set of complexes. Machine-learning SFs have indeed been implemented with as much training data as possible [16,17]. A different scenario, not considered by Li and Yang, would be to compare the SFs on a target with few training complexes (e.g., because the target is hard to co-crystallise with ligands). We think that the niche of classical SFs could be in this type of targets, as suggested by results with the smallest training sets in Figure 1. However, it should be noted that SFs do not only learn from complexes containing proteins with high global similarity (e.g., RF-Score excels at predicting the binding affinities of P38 kinase complexes when trained with complexes not including this target [18,19]). There are many reasons that could explain this behaviour, such as the fact that the same ligand–binding domain may be found in globally dissimilar proteins.

Lastly, regarding Claim D (the reason for RF-Score superiority remains unclear), we argue that the reason for the improvement of the scoring power made by machine-learning SFs has actually been quite clear since the original RF-Score was published [1]. Machine-learning algorithms are on average better at relating interatomic features characterising a complex with its binding affinity than the linear regression techniques employed by classical SFs [8]. Figure 1 and Figure 2 demonstrate this point: RF::X-Score outperforms X-Score using the same features and the full 1105 complexes for training (i.e., when both SFs only differ in the employed regression algorithm). The same has been shown for other classical SFs (e.g., AutoDock Vina [11] or Cyscore [13]). In supporting this claim, Li and Yang affirmed that machine-learning SFs have been found to have much worse virtual screening power than classical SFs [20], but this is factually incorrect. That study actually showed that the original version of RF-Score, which was designed for the scoring power, does not offer competitive performance in the related problem of virtual screening. Nevertheless, many machine-learning SFs have been found to have substantially better virtual screening power than classical SFs, before [4,5,21] and after [22,23,24,25,26] the cited study. In fact, when appropriate care is taken to tailor RF-Score for virtual screening, even a machine-learning SF with ultra-simple features (RF-Score-VS with v1 features) outperforms a range of classical SFs in terms of enrichment [26].

## 4. Materials and Methods

The protein–ligand complexes used in this study were the same as those in Li and Yang’s study [9], which were retrieved from the PDBbind v2007 refined set. The 195 diverse complexes in the core set were reserved for testing, and the rest 1105 complexes were subdivided into multiple nested training sets according to their pairwise structural and sequence similarity cutoffs. With such similarity matrices, we first confirmed the number of training samples given a certain cutoff was identical to the value listed in [9]. The smallest cutoff was 0.4 for structural similarity (corresponding to a training set of 116 complexes) and 0.3 for sequence similarity (corresponding to a training set of 181 complexes) in [9]. To see how the performance of the considered SFs varied when trained on fewer complexes, we added two new cutoffs of 0.35 and 0.3 for structural similarity (resulting in new training sets of just 43 and 9 complexes) and two new cutoffs of 0.25 and 0.2 for sequence similarity (resulting in new training sets of just 56 and 14 complexes). For X-Score, the four pre-calculated energy terms were obtained from Li and Yang. Rp values calculated by us were compared to their values listed in [9] and found to be identical (linear regression is a deterministic procedure). For the three machine-learning SFs, RF::X-Score retained the same four features (energy terms) as X-Score, RF-Score [1] used 36 intermolecular atomic distance counts as descriptors, and RF-Score-v3 [11] added six more descriptors from AutoDock Vina. Since these machine-learning SFs are stochastic, for each cutoff we built 10 instances and reported the average performance in Figure 1 and Figure 2. Instead of the cutoff values used by Li and Yang in the horizontal axis of their plots (Figure 2b), we used the number of training samples to evidence that machine-learning SFs only required a small part of the full training set to outperform X-Score (Figure 2a). The table listing the test set performance of SFs can be found at Supplementary Materials.

## 5. Conclusions

Unlike X-Score, the predictive performance of both versions of RF-Score improves when the complexes with most similar proteins to those in the test set are included in the training set. It is this capability that is actually necessary for real-world applications. The scoring power of machine-learning SFs has already been demonstrated in other studies to increase with larger training sets, which inevitably contain proteins with any degree of similarity to those in the test set. We have shown that machine-learning SFs do not only learn from complexes containing proteins with high global similarity. Hence, there is no support for the claim that the outstanding scoring power of RF-Score is exclusively due to a high number of globally similar proteins. On the other hand, the mathematical relationship between the atomic-level interactions and the binding affinities of the protein–ligand complexes is strongly nonlinear. Machine-learning algorithms are therefore expected to excel at this problem, and significant improvement has been seen after classical linear SFs, such as AutoDock Vina [11] and Cyscore [13], were modified to use random forest regression.

From a wider perspective, we think that this research topic would be better served by a non-competitive approach instead of the current machine-learning versus classical SFs trenches. As different targets are likely to be better predicted by different SFs, work intended to identify optimal SFs for each target and understand the reasons for such preferences is expected to be much more fruitful.

## Supplementary Materials

The following are available online at http://www.mdpi.com/2218-273X/8/1/12/s1, Table S1: test set performance of the scoring functions.

## References

[1] Ballester P.J., Mitchell J.B.O. (2010). A machine learning approach to predicting protein–ligand binding affinity with applications to molecular docking. Bioinformatics.

[2] Ashtawy H.M., Mahapatra N.R. (2015). A comparative assessment of predictive accuracies of conventional and machine learning scoring functions for protein–ligand binding affinity prediction. IEEE/ACM Trans. Comput. Biol. Bioinform..

[3] Zilian D., Sotriffer C.A. (2013). SFCscore(RF): A random forest-based scoring function for improved affinity prediction of protein–ligand complexes. J. Chem. Inf. Model..

[4] Li L., Wang B., Meroueh S.O. (2011). Support vector regression scoring of receptor–ligand complexes for rank-ordering and virtual screening of chemical libraries. J. Chem. Inf. Model..

[5] Ding B., Wang J., Li N., Wang W. (2013). Characterization of small molecule binding. I. Accurate identification of strong inhibitors in virtual screening. J. Chem. Inf. Model..

[6] Li H., Leung K., Wong M., Ballester P.J. (2016). Correcting the impact of docking pose generation error on binding affinity prediction. BMC Bioinform..

[7] Sun H., Pan P., Tian S., Xu L., Kong X., Li Y., Dan L., Hou T. (2016). Constructing and validating high-performance MIEC-SVM models in virtual screening for kinases: A better way for actives discovery. Sci. Rep..

[8] Ain Q.U., Aleksandrova A., Roessler F.D., Ballester P.J. (2015). Machine-learning scoring functions to improve structure-based binding affinity prediction and virtual screening. Wiley Interdiscip. Rev. Comput. Mol. Sci..

[9] Li Y., Yang J. (2017). Structural and sequence similarity makes a significant impact on machine-learning-based scoring functions for protein–ligand interactions. J. Chem. Inf. Model..

[10] Cheng T., Li X., Li Y., Liu Z., Wang R. (2009). Comparative assessment of scoring functions on a diverse test Set. J. Chem. Inf. Model..

[11] Li H., Leung K.-S., Wong M.-H., Ballester P.J. (2015). Improving AutoDock Vina using random forest: The growing accuracy of binding affinity prediction by the effective exploitation of larger data sets. Mol. Inform..

[12] Breiman L. (2001). Random forests. Mach. Learn..

[13] Li H., Leung K.-S., Wong M.-H., Ballester P.J. (2014). Substituting random forest for multiple linear regression improves binding affinity prediction of scoring functions: Cyscore as a case study. BMC Bioinform..

[14] Ballester P.J. (2012). Machine learning scoring functions based on random forest and support vector regression. Lect. Notes Bioinform..

[15] Li H., Leung K.-S., Wong M.-H., Ballester P. (2015). Low-quality structural and interaction data improves binding affinity prediction via random forest. Molecules.

[16] Pires D.E.V., Ascher D.B. (2016). CSM-lig: A web server for assessing and comparing protein–small molecule affinities. Nucl. Acids Res..

[17] Zilian D., Sotriffer C.A. (2013). Combining SFCscore with Random Forests leads to improved affinity prediction for protein–ligand complexes. J. Cheminform..

[18] Kramer C., Gedeck P. (2010). Leave-cluster-out cross-validation is appropriate for scoring functions derived from diverse protein data sets. J. Chem. Inf. Model..

[19] Ballester P.J., Mitchell J.B.O. (2011). Comments on “leave-cluster-out cross-validation is appropriate for scoring functions derived from diverse protein data sets”: Significance for the validation of scoring functions. J. Chem. Inf. Model..

[20] Gabel J., Desaphy J., Rognan D. (2014). Beware of machine learning-based scoring functions-on the danger of developing black boxes. J. Chem. Inf. Model..

[21] Durrant J.D., McCammon J.A. (2011). NNScore 2.0: A neural-network receptor–ligand scoring function. J. Chem. Inf. Model..

[22] Pradeep P., Struble C., Neumann T., Sem D.S., Merrill S.J. (2015). A novel scoring based distributed protein docking application to improve enrichment. IEEE/ACM Trans. Comput. Biol. Bioinform..

[23] Silva G.C., Simoes C.J.V., Carreiras P., Brito R.M.M. (2016). enhancing scoring performance of docking-based virtual screening through machine learning. Curr. Bioinform..

[24] Wang C., Zhang Y. (2017). Improving scoring-docking-screening powers of protein-ligand scoring functions using random forest. J. Comput. Chem..

[25] Pereira J.C., Caffarena E.R., dos Santos C.N. (2016). Boosting docking-based virtual screening with deep learning. J. Chem. Inf. Model..

[26] Wójcikowski M., Ballester P.J., Siedlecki P. (2017). Performance of machine-learning scoring functions in structure-based virtual screening. Sci. Rep..

